# Complete Genome Sequences of Eight Methicillin-Resistant Staphylococcus aureus Strains Isolated from Patients in Japan

**DOI:** 10.1128/MRA.01212-19

**Published:** 2019-11-21

**Authors:** Miyako Hikichi, Miki Nagao, Kazunori Murase, Chihiro Aikawa, Takashi Nozawa, Akemi Yoshida, Taisei Kikuchi, Ichiro Nakagawa

**Affiliations:** a Department of Microbiology, Graduate School of Medicine, Kyoto University, Kyoto, Japan; b Clinical Laboratory Medicine, Graduate School of Medicine, Kyoto University, Kyoto, Japan; c Laboratory of Genomics, Frontier Science Research Center, University of Miyazaki, Miyazaki, Japan; d Department of Infectious Diseases, Faculty of Medicine, University of Miyazaki, Miyazaki, Japan; Indiana University, Bloomington

## Abstract

Methicillin-resistant Staphylococcus aureus (MRSA) is a major pathogen causing nosocomial infections, and the clinical manifestations of MRSA range from asymptomatic colonization of the nasal mucosa to soft tissue infection to fulminant invasive disease. Here, we report the complete genome sequences of eight MRSA strains isolated from patients in Japan.

## ANNOUNCEMENT

Staphylococcus aureus is a causative agent that triggers a wide range of clinical infections, such as bacteremia, catheter-associated infections, and surgical site infections (1, 2). Among S. aureus strains, methicillin-resistant Staphylococcus aureus (MRSA) is a representative drug-resistant bacterium and one of the most successful modern pathogens, accounting for 60% of clinical S. aureus infections in Japan (3). Vancomycin and daptomycin are the agents of choice for acceptable treatment of invasive MRSA infections. Alternative agents that may be used for second-line or salvage therapy include telavancin, ceftaroline, and linezolid (4). However, it has recently been reported that acquisition of the *vanA* gene cluster during antibiotic therapy leads to resistance to vancomycin (5). Here, we report the complete genome sequence of eight MRSA strains isolated in Japan, which showed multidrug resistance and possessed related genes in their plasmids. This genome information could help understanding of the multidrug resistance machinery of S. aureus.

Eight MRSA strains were isolated from the specimens of patients during the period of 2004 through 2018 and identified according to CLSI approved standard M100. All clinical samples were cultured on sheep blood agar, chocolate agar, and Drigalski agar at 35°C for 24 h, and the colonies were selected and then identified using matrix-assisted laser desorption ionization–time of flight mass spectrometry (MALDI-TOF MS). The Kyoto University institutional review board approved the protocol for use of the clinical data and strains.

Eight MRSA strains were incubated for 15 h at 37°C in Trypticase soy medium. The bacterial cells were lysed with lysostaphin and lysozyme, and then the genomic DNA was extracted by the phenol-chloroform extraction method. Extracted DNA was sequenced by a combination of short-read and long-read sequencing platforms. An Illumina short-read library was prepared using a Nextera DNA library prep kit, and paired-end reads were generated using a MiSeq reagent kit (v3-600) on the MiSeq platform (Illumina). A MinION long-read library was prepared from unsheared genomic DNA using the Rapid Barcoding kit (catalog number SQK-RBK004) and sequenced with an R9 flow cell (FLO-MIN106) on a MinION device (Oxford Nanopore Technologies). The Illumina data were preprocessed using Trimmomatic (v0.36) to remove adapter and low-quality sequences (6), and hybrid assembly with the MinION long reads was performed using the Unicycler pipeline (v0.4.7b) with default parameters (7). Next, the assembled genome sequence was annotated using Prokka (v1.11) with default settings (8). Assembly statistics, general genome information, and relevant characteristics are summarized in Table 1.

**TABLE 1:** Assembly statistics, general genome information, and relevant characteristics of eight MRSA strains

Statistic or characteristic	Data for MRSA strain^*b*^:							
	KUN1163	KUH140013	KUH140046	KUH140087	KUH140331	KUH180038	KUH180062	KUH180129
Strain information								
Origin	Sputum	Sputum	Nasal cavity	Blood	Blood	Pus	Sputum	Blood
Clinical symptom	Colonization	Pneumonia	Colonization	Sepsis	Sepsis	Skin and soft tissue infection	Colonization	Sepsis
Yr of isolation	2014	2014	2014	2014	2014	2018	2018	2018
Antibiotic resistance (MIC [μg/ml])^*a*^	CEZ (≥64), IPM (≥32), LVFX (≥16), GM (≥32), EM (≥16), CLDM (≥16), MPIPC (≥16)	CEZ (≤8), IPM (2), LVFX (>4), GM (>8), EM (>4), CLDM (>2)	CEZ (>16), IPM (8), LVFX (>4), GM (>8), EM (>4), CLDM (>2), MPIPC (>2)			CEZ (16), IPM (8), LVFX (>4), GM (>8), EM (>4), CLDM (>2), MPIPC (>2)	CEZ (>16), IPM (>8), LVFX (>4), GM (>8), EM (>4), MINO (>8), CLDM (>2), MPIPC (>2)	
Assembly and genome statistics								
No. of reads								
MinION	91,288	79,719	88,426	120,397	56,527	100,511	108,241	70,353
MiSeq	451,782	1,059,412	904,752	810,867	1,021,192	876,198	891,514	749,820
Total no. of bases								
MinION	621,945,741	582,298,636	654,264,184	578,007,623	379,853,580	811,349,149	815,396,551	490,139,316
MiSeq	186,281,854	415,398,345	375,301,178	334,508,054	399,768,647	349,878,661	354,906,810	323,657,748
Coverage (×)	274.46	351.62	360.06	330.43	283.05	406.50	394.08	280.88
Chromosome description								
Genome size (bp)	2,914,567	2,804,820	2,825,163	2,761,631	2,751,401	2,822,409	2,939,465	2,876,735
G+C content (%)	32.91	32.78	32.78	32.42	32.85	32.78	32.89	32.95
No. of CDSs^*c*^	2704	2560	2588	2511	2513	2584	2724	2682
No. of rRNAs	5	6	6	6	6	6	5	5
No. of tRNAs	61	62	60	61	61	62	59	61
No. of tmRNAs^*d*^	1	1	1	1	1	1	1	1
GenBank accession no.	AP020324	AP020311	AP020313	AP020315	AP020316	AP020318	AP020320	AP020322
Plasmid description								
Genome size (bp)	30,220	32,574	34,268		2,987	34,268	30,217	20,547
G+C content (%)	29.15	28.72	29.36		29.53	29.36	29.16	29.04
No. of CDSs	35	45	40		3	41	35	24
GenBank accession no.	AP020325	AP020312	AP020314		AP020317	AP020319	AP020321	AP020323

a MIC values (μg/ml) were determined by the broth microdilution method approved by CLSI and are shown in parentheses next to antibiotics.

b CEZ, cefazolin; IPM, imipenem/cilastatin; LVFX, levofloxacin; GM, gentamicin; EM, erythromycin; MINO. minocycline; CLDM, clindamycin; MPIPC, oxacillin.

c CDSs, coding sequences.

d tmRNAs, transfer-messenger RNAs.

Among the eight MRSA genomes, seven strains (excluding strain KUH140087) have a 3- to 30-kb plasmid carrying similar antimicrobial or antiseptic resistance genes [*aac(6′)-Ie*, *aph(2″)-Ia*, *qacA*, *qacB*, *aph(2″)-If*, *fosD*, and *blaZ*] in different combinations. It has been reported that genes for drug resistance are often derived from plasmids (9, 10), suggesting the importance of the investigation of antibiotic resistance plasmids for understanding S. aureus infection and its future treatments.

### Data availability.

The complete genome sequences of eight MRSA strains in this study have been deposited in DDBJ/EMBL/GenBank under accession numbers AP020311 to AP020325. The raw Illumina and MinION read data can be found in the DDBJ Sequence Read Archive/NCBI SRA under accession number DRA008776.
